# Effects of Self-Expressive Brand and Susceptibility to Interpersonal Influence on Brand Addiction: Mediating Role of Brand Passion

**DOI:** 10.3389/fpsyg.2021.602023

**Published:** 2021-02-05

**Authors:** Shizhen Bai, Yue Yin, Yubing Yu, Sheng Wei, Rong Wu

**Affiliations:** ^1^School of Management, Harbin University of Commerce, Harbin, China; ^2^Logistics and E-commerce College, Zhejiang Wanli University, Ningbo, China

**Keywords:** susceptibility to interpersonal influence, self-expressive brand, consumer-brand relationship, brand passion, brand addiction

## Abstract

Although the concept of the consumer–brand relationship has undergone rapid change over the past two decades, the issue of brand addiction is still generally neglected in the literature. Based on social identity theory, the research develops a conceptual model of the influence of self-expressive brands (SEBs) and susceptibility to interpersonal influence (SUSCEP) on brand addiction. The results of this research demonstrate both separate and joint effects of SEBs and SUSCEP on brand addiction. In addition, harmonious brand passion and obsessive brand passion positively mediate the relationships among SEB, SUSCEP, and brand addiction. The research explores the formation mechanism of brand addiction from a new perspective and has important practical implications for brand marketers concerned with finding the most effective means to enhance the consumer–brand relationship.

## Introduction

The concept of the customer–brand relationship initially focused on general attitudinal tendencies like commitment and loyalty, but researchers have expanded the concept to examine more intense forms of brand connection, such as brand love (Batra et al., 2012; Bagozzi et al., 2017), brand attachment (Jahn et al., 2012; Dolbec and Chebat, 2013), and, most recently, brand addiction (Cui et al., 2018; Mrad et al., 2020). The literature on brand addiction is still relatively nascent, and researchers are mainly concerned with its operationalization and conceptualization (e.g., Mrad and Cui, 2017, 2020). Because brand addiction represents the most intense or closest level of relation with brands, it is becoming a popular topic of study in brand marketing. However, given that brand addiction can lead to positive or negative outcomes (e.g., Mrad and Cui, 2017; Mrad et al., 2020), there is a strong impetus to understand its antecedents.

Swimberghe et al. (2014) proposed two broad categories of antecedents to an individual’s behavior: the self and the influence of others. There is limited empirical research that takes both categories into consideration. Some studies have approached the issue of brand relationships from the self-identity perspective; these include Wallace et al. (2014), who explored the association between SEBs and brand love; Lee and Workman (2015), who attempted to identify the role of self-expressive brands in young consumers’ brand relationships. Other research has highlighted the influence of others by discussing the role that consumption plays in bringing people together—i.e., the ‘linking value’ of brand consumption (Cova, 1997). So far, there is no research that can fully prove how brand addiction is separately and jointly influenced by factors related to these two categories (i.e., the self and the influence of others). Identifying these factors is expected to help brand managers choose more appropriate ways to attract consumers and facilitate close and intense brand relationships. We try to highlight on the issue by exploring the effects of both the self and the influence of others on brand addiction.

Mrad and Cui (2017, 2020) developed a definition of brand addiction and began to explore its antecedent variables from different perspectives. At present, the factors known to influence brand addiction mainly relate to attitude (e.g., brand trust, brand attachment), perceived value, individual traits (e.g., perfectionism, self-enhancement), interpersonal influences, transcendent consumer experiences, and brand characteristics. Following Swimberghe, we consider antecedents related to customers’ affective connections with brands (e.g., brand self-expression and brand passion), as these closely reflect customer commitment and have clear implications for brand addiction (Fournier, 1998; Albert et al., 2013). In addition, susceptibility to interpersonal influence can affect customers’ brand choices (Ruane and Wallace, 2015). Indeed, the research has shown that in shaping customer attitude and behavior, the susceptibility to interpersonal influence has a role that cannot be ignored, and the greater the impact of interpersonal, the more likely consumers are to have strong feelings toward brands (Swimberghe et al., 2014).

Drawing on the insights above, our research explores how customers’ affective connections with brands (i.e., brand self-expression and brand passion) and the influence of others affect brand addition. In a competitive landscape characterized by a large number of similar brands and products (Hwang and Kandampully, 2012), marketing strategies focused on price reductions and loyalty plans may be insufficient. A more promising strategy may be to help customers form intense brand connections, as strong consumer–brand relationships are believed to be the driving force in creating more sustainable brands (Park et al., 2006). Therefore, our research explores how customers’ self-expression through brands and susceptibility to interpersonal influence affect their brand relationships and addictive behaviors. By examining the roles that the self and the influence of others play in this process, the results may help to deepen the understanding of brand addiction and have practical implications for brand marketers seeking efficient ways to enhance the consumer–brand relationship.

The rest of the study is organized as follows. In the second part, conceptual framework and theoretical background are presented. The third part focuses of hypotheses development. The fourth part describes the methodology, including the measures, data collection procedure, and sample. The fifth part estimates and assesses the structural model, evaluates the competitive models, and tests the mediating effects. The sixth part presents the results and primary conclusions, which includes implications for the literature and practitioners, limitations, future research direction of this research topic.

## Conceptual Framework and Theoretical Background

Social identity theory is considered appropriate for exploring the psychological mechanism of brand addiction, since identification has significant contribution to maintain the consumer–brand relationship (Lam et al., 2010). The social identity theory was developed by Tajfel and Turner who defined it “as that a part of an individual’s self-concept which comes from the knowledge of his membership of social groups as well as the value and emotional significance attached to the membership” (Tajfel and Turner, 1979). The theory is usually associated with the studies of self-concept, and explains consumer behavior based on the interaction between self and society (Lam et al., 2010). On the one hand, the theory explains why consumers arouse strong feeling toward certain brands: consumers are eager to express their identities and differentiate themselves through certain brands. On the other hand, certain brands are consumed with the goal of gaining positive social recognition from groups to which customers hope to belong (Chernav et al., 2011; Sreejesh et al., 2016).

In addition, this study takes into account the psycho-social signals of “influence,” which could constitute a theoretical basis of explanation of the binomial self/others (Poggi and D’Errico, 2012; Papapicco and Mininni, 2020). As Bearden and co-workers suggest, there is no adequate understanding of consumer behavior without considering the impacts of interpersonal influence on the individual’s attitude, value, or consumption decision. Influence can be goal-directed, and occasionally individuals influence each other inadvertently (Poggi and D’Errico, 2012). The reason why consumers buy brand products is not only to meet the expectations of others, but also to send a signal to the consumer groups that they want to be recognized (Chernav et al., 2011).

**FIGURE 1:**
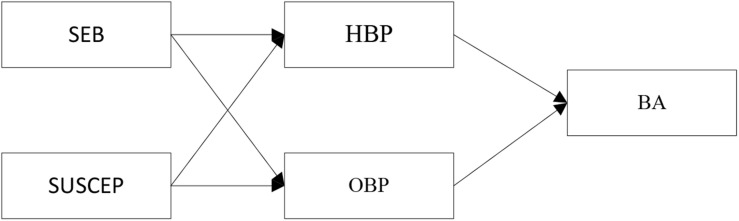
Conceptual model. SEB, Self-expressive brand; SUSCEP, Susceptibility to interpersonal influence; HBP, Harmonious brand passion; OBP, obsessive brand passion; BA, Brand addiction.

This study explores the formation mechanism of brand addiction from the perspectives of the self and society. Based on previous research (Swimberghe et al., 2014), the constructs of self-expressive brands (SEBs) and susceptibility to interpersonal influence (SUSCEP) are used to reflect these two aspects. In addition, the shaping of the individual or social self may be autonomous or controlled in nature, and the resulting brand passion may be harmonious or obsessive. Considering the above, we investigate the mediating roles of harmonious brand passion (HBP) and obsessive brand passion (OBP) in the relationships among SEB, SUSCEP, and brand addiction to elucidate the process by which SEB and SUSCEP translate into brand addiction. The conceptual model guiding this research is outlined in Figure 1. The hypotheses are developed in the following sections.

## Research Hypotheses

### Self-Expressive Brands

An important part of the consumer–brand relationship is the idea of SEBs (Lee and Workman, 2015), which are defined as “the consumer’s perception of the degree to which the specific brand enhances one’s social self and/or reflects one’s inner self” (Carroll and Ahuvia, 2006). To be specific, focus on personal self-concept, and the better ways to express themselves by brands (Loureiro et al., 2010). It is not difficult to find that the brand of self-expression is a symbol of personal achievement and a necessary carrier of social integration, which helps consumers better express their personal needs (Ruane and Wallace, 2015). Because brands that are more self-expressive tend to be more loved, managers can elicit more intense emotional responses from consumers by enhancing this aspect of their offerings. In addition, as consumers feel more love toward brands which are able to help them to shape their identity and self-expressiveness, it follows that SEBs should be one of the motivators of brand addiction (Mrad et al., 2020). A brand’s self-expressive benefits can facilitate addiction, and a consumer–brand relationship can be maintained by enhancing the potential for self-expression (Ruane and Wallace, 2015).

The social identity theory suggests that individuals hope to express their own identities and differentiate themselves through certain brands. The existence of these brands provide a good way for individuals to achieve self-expression (Aaker, 2009), and their advantages also stimulate consumers’ purchasing behavior. Combined with past research, it can be seen that customers who consume self-expression brands are more dependent on these brands than other customers (Ruane and Wallace, 2015). This conclusion is consistent with the argument of Liu et al. (2012). In short, when consumers think that the brand and their perceived self-image are the same, their trust and loyalty to the brand will increase. Most customers prefer to choose brands that match their own image or can symbolize their personal identity, and such brands are expected to elicit stronger emotional responses from consumers. In accordance with Aaker (2009), self-expressive brands can deepen the relationships between brands and consumers to a certain extent, and have also established that SEBs are an important factor affecting brand passion (Bauer et al., 2007).

Reviewing the literature related to marketing, it is found that there are many academic studies on brand passion (Swimberghe et al., 2014; Das et al., 2019; Mukherjee, 2019; Pourazad et al., 2019), and researchers have made corresponding definitions based on different backgrounds. The concepts have already accepted the explanation of brand passion by Swimberghe et al. (2014). They believe it reflects consumer’s feeling for brands, which he/she values, blends into his/her identity, and invests resources. The definition assumes that there are close theoretical relationships between brand passion and self-identity, which also proves that the perspective of consumers’ brand passion is consistent with the previous articles (Albert et al., 2013). There are two types of brand passion: harmonious and obsessive (Swimberghe et al., 2014). The difference lies in how a brand is internalized in a person’s identity. When a brand projects an individual’s identity through self-directed internalization, HBP gradually forms (Swimberghe et al., 2014). When a brand shows his/her identity through controlled internalization, OBP may result. The perception that a brand may enhance a consumer’s identity generates positive emotions, leading to brand passion. Therefore, we propose:

H1. The level of SEB has a positive impact on HBP.

H2. The level of SEB has a positive impact on OBP.

### Susceptibility to Interpersonal Influence

One of the most significant factors that determine an individual’s behavior is the impact of others (Swimberghe et al., 2014). When consumers’ behavior can be observed, these effects will emerge, and consumers’ interpersonal influence will be directly reflected in their purchase behavior (Goudge et al., 2017). According to Bearden et al. (1989), SUSCEP is reflective of personality characteristics, which not only embodies certain need of a consumer to improve his/her own image through purchasing brand products, but also shows the willingness to comply with other people’s expectations of purchasing decisions. Consumers who have a high SUSCEP are expected to prefer brands that manifest desirable traits for others to see Lertwannawit and Mandhachitara (2012), and have a stronger emotional response to the brands that symbolize their personal images (Astakhova et al., 2017). Mrad et al. (2020) showed that addiction to certain fast-fashion and luxury brands was associated with consequences related to interpersonal relations and financial issues.

When people consciously adjust their own deeds and beliefs to achieve consistency with others, the susceptibility is fairly obvious (Nabi et al., 2019). According to the psycho-social signals of “influence”, the reason why consumers buy brand products is not only to meet the expectations of others, but also to send a signal to the consumer groups that they want to be recognized (Chernav et al., 2011; Sreejesh et al., 2016). Thus, a considerable number of consumers will show their group membership by purchasing some brand products. Individuals with higher susceptibility are also more willing to use interpersonal search to obtain the information they want to know (Mourali et al., 2005). As long as their information needs are met, the information will be regarded as real and can be used to meet the expectations of others (Kiani and Laroche, 2019). The consumers that desire to be recognized in this manner also expects to establish a good connection with society (Lertwannawit and Mandhachitara, 2012). Inner satisfaction is conducive to the autonomous internalization process of consumer–brand identification and the generation of HBP.

As far as consumers are concerned, the others’ influence is mainly from colleagues, family members, and friends. The disapproval of their specific purchase behavior by these reference groups will bring great psychological pressure to consumers and result in their blind pursuit of psychological convergence (Sharma and Klein, 2020). In this case, consumers buy brands because of interpersonal pressure, resulting in a brand relationship that is not controlled by the individual. This can lead to OBP, which originates from the controlled internalization process of consumer–brand identification (Das et al., 2019). Combined with existing research data, we can see that the impact of SUSCEP on OBP has been verified (Swimberghe et al., 2014), which also shows a consumer who is highly sensitive to interpersonal influences will be apt to have an obsessive passion for brands. Therefore, we propose:

H3. The level of SUSCEP has a positive impact on HBP.

H4. The level of SUSCEP has a positive impact on OBP.

### The Mediating Role of HBP and OBP

The relationships between consumers and brands are usually closely related to the personality of consumers and the ways in which their interpersonal relationships form. When consumers have a high degree of recognition and trust for a certain brand, their emotion will be very positive. Social identity theory has been considered appropriate for understanding customers’ psychological mechanisms (Lam et al., 2010), and the recognition effect can supplement other research topics, such as customer brand relationship (Swimberghe et al., 2014). The theory holds that consumers shape their private or social selves to define their self-concepts. Brands contribute to the private/social self construction on account of consumers regarding the brand as a medium to gain identity, which can be recognized by customers (Lam et al., 2010). The shaping of the individual and social roles may be autonomous or controlled, generating two forms of brand passion, i.e., HBP or OBP (Vallerand et al., 2003). Both of them effectively prove how a brand internalizes a person’s identity.

Under the influence of various schemas, brand passion can realize the close relationship between brand and customers, and customers’ demand for identity will last for a long time (Schmalz and Orth, 2012). HBP provides customers an internal motivation to lend importance to a brand, desire to get it without any contingencies or other influences (Albert et al., 2013). In the case of OBP, however, customers are compelled to purchase a brand due to interpersonal (social) or intra-personal (internal) pressures (Rauschnabel and Ahuvia, 2014), and this tends to lead to brand obsession and culminate in brand addiction (Fournier, 1998). Some authors consider the consumer-brand relationships are continuous points, and the relationships increase step by step (e.g., Fournier, 1998; Veloutsou and Moutinho, 2009). Thus, emotional passion toward a brand is a predictor of addiction.

Considering the continuum of consumer-brand relationships, we propose that brand passion is an element that ought to be taken into account. Based on the mediational hypothesis route, assuming that consumers regard a brand as one of their relationship partners, several brand clues that meet their own needs can help them to define themselves relatively accurately and to achieve consistency with others; this, in turn, triggers a strong emotional reaction in the form of brand passion, either harmonious or obsessive (Das et al., 2019). In other words, SEBs and SUSCEP may be considered important drivers of brand passion, which, in turn, leads to a higher tendency toward brand addiction. Accordingly, we hypothesize that SEBs and SUSCEP influence brand addiction through HBP and OBP. Thus, we propose:

H5. HBP mediates the effect of both SEB and SUSCEP on brand addiction.

H6. OBP mediates the effect of both SEB and SUSCEP on brand addiction.

## Research Methodology

### Ethics Approval Statements

The studies involving human participants were reviewed and approved by University of Commerce. All participants were consumers familiar with a certain brand finding through Harbin Mingyue Market Research Consulting Co., Ltd. using questionnaire. They verbally agreed to participate in this study and returned the questionnaire within a fixed time.

### Data Collection and Sample

In the study, consumers with strong feelings about a particular brand were found through the snowball recommendation method. Respondents were asked to select favorite brands and fill in a questionnaire. Although snowball sampling is a non-random sampling process, this method significantly increases the likelihood of finding target consumers. There is evidence that its use can help researchers obtain high-quality and reliable data. A total of 800 questionnaires were distributed during the study period. 526 valid questionnaires were obtained after eliminating the questionnaires with missing value, contradictory answers. The detailed list of the demographic information was showed in Table 1.

**TABLE 1:** Demographic information of respondents.

Criteria	Number	Percentage (%)
***Sex***		
Male	200	38.0%
Female	326	62.0%
***Age***		
18–25	261	49.6%
26–35	121	23.0%
36–45	114	21.7%
> 45	30	5.7%
***Education***		
Junior high school or lower	8	1.5%
High school/technical school	20	3.8%
Graduate	351	66.7%
Master’s degree or higher	132	25.1%
Ph.D.	15	2.9%
***Occupation***		
Student	249	47.3%
Government or public institution employees	40	7.6%
Enterprise staff	121	23.0%
Self-employed	102	19.4%
Others	14	2.7%

The sample consisted of consumers with strong feelings about a particular brand in China, ranging in ages from 18 to 55 (*M* = 34.88; *SD* = 8.237). Among the respondents, 62.0% were female, and 38.0% were male. About 47.3% of the respondents were students, 7.6% were employees of the government or public institutions, 23% were employees of enterprises, 19.4% were self-employed, and 2.7% were employed in other occupations.

### Measures

Reliability analysis showed that Cronbach’s alpha values were all above 0.70: SEB (0.88), SUSCEP (0.73), HBP (0.76), OBP (0.90), and brand addiction (0.88). Consequently, we preliminarily believes that the measurement values selected in the study have certain reliability.

The measurements of the constructs were validated based on previous research, which could be seen in Table A1. Regarding SEB, the eight-item scale of Carroll and Ahuvia (2006) was used; this included items such as “this brand reflects my personality” and “this brand is an extension of my inner self.” The SUSCEP measures were taken from Lertwannawit and Mandhachitara (2012) as well as Ruane and Wallace (2015); these included items such as “I achieve a sense of belonging by purchasing the same products and brands that others purchase” and “I often identify with other people by purchasing the same products and brands they purchase.” Regarding HBP and OBP, the measures of Swimberghe et al. (2014) were adopted; these included items such as “this activity allows me to live a variety of experiences” and “the urge is so strong that I can’t help myself from doing this activity.” Brand addiction was measured using the scales of Mrad and Cui (2017), with such statements as “I often find myself thinking about my favorite brand” and “I follow my favorite brand’s news all the time.” For the above measures, a 5-point Likert-type scale (1 = strongly disagree; 5 = strongly agree) was used.

## Data Analysis and Results

### Measurement Model

Items that correlated poorly with other items in all scales were detected by conducting a preliminary data analysis. As a result, one item of the original HBP scale and two items of the original brand addiction scale were deleted. A confirmatory factor analysis (CFA) was implemented for evaluating the measurement model’s performance. The maximum likelihood (ML) estimation strategy was used to perform the estimation. This strategy assumes the multi-normality of the observed variables’ distribution.

As advised by Kline (2017), kurtosis and skewness were evaluated to examine the departure from normality. Kurtosis in the data ranged from −1.19 to 1.8, and skewness ranged from −1.07 to 0.23. Thus, considering the thresholds (kurtosis < 20.0; skewness < 3.0) put forward by Kline (2017), the items met the assumptions of the ML estimation strategy. In spite of the results show that it is inconsistent with multiple normality, the application of ML estimation strategy can effectively solve this problem. Especially kurtosis needs to be tested because it has a large influence on the covariance and variance, which are the foundation of structural equation modeling. However, in accordance with the simulation research proposed by West et al. (1995), only the kurtosis value exceeds 7 is indicative of a serious departure from normality.

The model’s fit indices acted well in terms of the acceptable thresholds in the core literature (Hair et al., 2006; Hwang and Kandampully, 2012). Although the chi-square (χ^2^) test yielded a result of 708.466 with *df* = 199, which was statistically significant (*p* < 0.01), the remainder of the fit indices—i.e., the goodness of fit index (GFI; 0.88), the root mean square error approximation (RMSEA; 0.07), the comparative fit index (CFI; 0.91), the Tucker-Lewis index (TLI; 0.89), the incremental fit index (IFI; 0.91), and the normed fit index (NFI; 0.88)—implied that the measurement model was able to be accepted.

The standardized factor loadings were relatively large (every loading was over the 0.5 threshold) and statistically significant (*p* < 0.01). In addition, since R^2^ values are all above the threshold value of 0.20, individual items are credible, and the convergence effectiveness of the above measures is also proved. By analyzing Table 2, we can clearly see that the comprehensive reliability of all the scales is greater than 0.70, indicating that the scales were internally consistent. For most of the structures in the model, the extracted average variance is not only greater than 50%, but also larger than the square of the correlation coefficients of the two potential variables. These results indicate the model’s discriminant validity (see Table 3). Given the high correlation between HBP and brand addiction (*r* = 0.72), an alternative four-element model where brand addiction and HBP were loaded on a single element was also tested. This model yielded a worse fit (χ^2^ = 848.270, *df* = 203, *p* < 0.001, RMSEA = 0.08, CFI = 0.88, TLI = 0.87, IFI = 0.88, NFI = .85, GFI = 0.86; Δχ^2^ = 139.804, Δ*df* = 4, *p* < 0.001), implying that HBP and brand addiction are distinct. In conclusion, the constructs present acceptable levels of discriminant validity, convergent validity, and reliability.

**TABLE 2:** Confirmatory factor analysis results.

Construct	Dimension/Items	Stand. Loadings	*t*-value	CR	AVE
Self-expressive brand (SEB)	Inner self	0.82	–	0.79	0.66
	Social self	0.80	18.34		
Susceptibility to interpersonal influence (SUSCEP)	I achieve a sense of belonging by purchasing the same products and brands that others purchase.	0.70	–	0.73	0.47
	If I want to be like someone, I often try to buy the same brands that they buy.	0.61	10.22		
	I often identify with other people by purchasing the same products and brands they purchase.	0.74	11.63		
Harmonious brand passion (HBP)	This activity allows me to live a variety of experiences.	0.60	–	0.74	0.42
	The new things that I discover with this activity allow me to appreciate it even more.	0.68	11.60		
	This activity allows me to live memorable experiences.	0.67	11.08		
	This activity reflects the qualities I like about myself.	0.63	10.54		
Obsessive brand passion (OBP)	I cannot live without it.	0.77	–	0.90	0.65
	The urge is so strong, I can’t help myself from doing this activity.	0.76	18.27		
	I am emotionally dependent on this activity.	0.81	18.94		
	I have a tough time controlling my need to do this activity.	0.87	20.15		
	I have almost an obsessive feeling for this activity.	0.82	18.81		
Brand addiction (BA)	I often find myself thinking about my favorite brand.	0.67	–	0.86	0.43
	I tend to give up some life activities and duties, such as the occupational, academic, and familial, to fulfill some activities related to my favorite brand.	0.60	12.28		
	I tend to allocate a certain portion of my monthly income to buy the products of my favorite brand.	0.61	12.53		
	I usually remember tenderly the previous experiences with my favorite brand.	0.68	13.80		
	I experience a state of impatience immediately before I can get hold of the products of my favorite brand.	0.61	12.48		
	I follow my favorite brand’s news all the time.	0.67	13.51		
	I usually plan when the next purchase of my favorite brand will be.	0.70	14.05		
	I would invest my money in some way to support my favorite brand.	0.72	14.48		

**TABLE 3:** Correlations and square root of AVE.

Construct	X_1_	X_2_	X_3_	X_4_	X_5_
SEB(X_1_)	***0.81***				
SUSCEP(X_2_)	0.76	***0.69***			
HBP(X_3_)	0.70	0.60	***0.65***		
OBP(X_4_)	0.69	0.59	0.55	***0.81***	
BA(X_5_)	0.77	0.65	0.72	0.72	***0.66***

*The bold italic numbers in diagonal are the square roots of the average variances extracted.*

*SEB, Self-expressive brand; SUSCEP, Susceptibility to interpersonal influence; HBP, Harmonious brand passion; OBP, Obsessive brand passion; BA, Brand addiction.*

### Structural Model

The model (shown in Figure 1) illustrates how both HBP and OBP mediate the impact of SEB and SUSCEP on brand addiction. It has been suggested that residuals linked to mediators be allowed to co-vary (Preacher and Hayes, 2008; Augusto and Torres, 2018). According to Gudmundsdottir et al. (2004), the residual correlation among mediators usually plays a substantive role. Thus, having the residuals of the mediator be steady at 0 is an unreasonable constraint that will cause the model to be misspecified. It was concluded that the mediators’ errors were correlated by adhering to the recommendation applied in previous studies (e.g., Augusto and Torres, 2018).

The hypotheses and results of the structural model are presented in Table 4. The chi-square (χ^2^) test yielded a value of 695.021 with *df* = 196 and *p* < 0.01. Moreover, the remaining fit indices implied a nice model fit (RMSEA = 0.07, CFI = 0.91, TLI = 0.89, IFI = 0.91, NFI = 0.88, GFI = 0.90). The results of the fit indices provide support to all the hypotheses. Meanwhile, the results indicate SEB has a stronger impact than SUSCEP on both HBP and OBP, while OBP and HBP have similar impacts on brand addiction.

**TABLE 4:** Results of the structural model.

Path	Stand. coeff.	*t*-value	*p*-value	Hypotheses
SEB→HBP	0.68	8.74	***	H1(+): S
SEB→OBP	0.68	10.66	***	H2(+): S
SUSCEP→HBP	0.25	3.65	***	H3(+): S
SUSCEP→OBP	0.25	4.15	***	H4(+): S
HBP→BA	0.48	6.73	***	H5(+): S
OBP→BA	0.51	8.55	***	H6(+): S

Stand. coeff., standardized coefficient; SEB, Self-expressive brand; SUSCEP, Susceptibility to interpersonal influence; HBP, Harmonious brand passion; OBP, Obsessive brand passion; BA, Brand addiction; S, supported.R^2^: HBP: 0.52; OBP: 0.52; BA: 0.74.***p < 0.001.

### Testing for Mediation Effects

We tested the mediating roles of OBP and HBP on the relationship between the dependent variable (brand addiction) and the independent variables (SUSCEP and SEB), three extra models were evaluated following the method used by James et al. (2006), Saenz et al. (2014), Augusto and Torres (2018), among others. The results of all four models are shown in Table 5. Model 1 is the base model. In Model 2, only the direct effects of SEB and SUSCEP on brand addiction were evaluated. Model 3 examined the direct impacts of SUSCEP and SEB on the dependent variable (brand addiction) and included the mediators. Finally, Model 4 corresponds to the base model, plus the direct effects of SEB and SUSCEP on brand addiction.

**TABLE 5:** Results of model estimation.

	Model 1, full mediation	Model 2	Model 3, non mediation	Model 4, partial mediation
SEB→HBP	0.68***		0.73***	0.67***
SEB→OBP	0.68***		0.75***	0.68***
SUSCEP→HBP	0.25***		0.19**	0.21**
SUSCEP→OBP	0.25***		0.21***	0.23***
SEB→BA	–	0.69***	0.82***	0.20*
SUSCEP→BA	–	0.27***	0.27***	0.15**
HBP→BA	0.48***		–	0.34***
OBP→BA	0.51***		–	0.40***
***R*^2^**				
HBP	0.52		0.57	0.49
OBP	0.52		0.61	0.51
BA	0.74	0.55	0.74	0.72

*p < 0.05; **p < 0.01; ***p < 0.001.

For the sake of verifying the existence of mediating effects, certain conditions must first be met. First, it is necessary for the independent variables to directly influence the mediators. Second, it is necessary for the mediators to directly affect the dependent variable. According to the evaluation result of model 1, the above two conditions are verified effectively. Third, it is necessary for the independent variable to directly affect the dependent variable with the absence of mediators. This condition was tested by an estimation of Model 2. The direct impacts of SUSCEP and SEB on brand addiction are important in the model. Finally, if the previous hypothesis turns out not to be supported after the mediators are included in the framework in the fourth step, or if their influence still exists but decreases, it indicates that there is partial mediation or full mediation. The results regarding mediating effects, which are shown in Tables 5, 6 indicate that Model 1 is a much nicer fit than Model 3 (non-mediation model) (Δχ^2^ = 44.906, Δ*df* = 0, *p* < 0.01). Furthermore, compared with Model 1, Model 4 (a partial mediation model) is a nicer fit (Δχ^2^ = 1.6, Δ*df* = 2, *p* < 0.01). In conclusion, given that the paths from SUSCEP and SEB to brand addiction still existed, but with reduced impacts, after including OBP and HBP, the partial mediation model was supported.

**TABLE 6:** Results of model comparison.

	χ^2^	*df*	Δ*df*	Δχ^2^	GFI	NFI	IFI	TLI	CFI	RMSEA
Model 1	685.021	196	–	–	0.90	0.88	0.91	0.89	0.91	0.07
Model 3	729.927	196	0	44.906	0.89	0.87	0.90	0.89	0.90	0.07
Model 4	683.426	194	2	1.6	0.90	0.88	0.91	0.89	0.91	0.07

## Discussion

This research investigated the effects of self-expressive brands and susceptibility to interpersonal influence on brand addiction, considering harmonious brand passion and obsessive brand passion as mediators. Companies seek to create highly emotional consumer–brand relationships (Schmid and Huber, 2019), but satisfying consumer wants and needs may not be enough; Moreover, company should make accurate market positioning for its brands, so as to meet the demands of customers’ life, and become an indispensible tool for customers to express themselves. Our findings indicate that SEBs have positive effects on both HBP (H1) and OBP (H2). These findings are consistent with prior studies that identified SEBs as a core contributor to customer–brand relationship enhancement (e.g., Swimberghe et al., 2014).

This research also finds that SUSCEP has positive effects on OBP and HBP (H3 and H4). These results explain the importance of interpersonal effects, which should be one of the motivators of consumer’s feeling for brands. The findings deepen understanding of the interplay among the constructs and indicate that SUSCEP might positively affect two forms of brand passion, meaning that a consumer who is sensitive to interpersonal influences can generate a harmonious or obsessive passion for brands.

At last, the results shows that the impacts of SUSCEP and SEBs on brand addiction are mediated by OBP and HBP. For brand managers, this implies a need to stimulate positive responses in terms of SUSCEP and SEBs, as this leads to brand passion and in turn has significant effects on brand addiction (H5 and H6). In line with Hatfield (1988), we support that passion relate to more intense emotional responses; emotional passion toward a brand is the antecedent of brand addiction. Moreover, brand managers should spend resources on a “passion branding” strategy and recognize the distinction between the two kinds of brand passion.

### Theoretical Implications

First, we explored the effects of SEBs and SUSCEP on brand addiction through quantitative research methods. The literature on brand addiction focuses on its conceptualization, trying to distinguish the following concepts, such as compulsive purchase or other forms of consumer-brand relationship (e.g., Mrad and Cui, 2017, 2020). Nevertheless, most of the related studies are qualitative in nature. For instance, Cui et al. (2018) analyzed 11 salient features of brand addiction by using qualitative data from projective interviewing and focus groups, and Mrad et al. (2020) did in-depth interviews to examine the motives behind consumers’ addictive behavior. The results extend current research by responding to the call of Mrad and Cui (2017) for a quantitative study to complement their findings.

Second, our research provides a new perspective for exploring the formation mechanism of brand addiction. In the light of social identity theory, the research finds SEBs, SUSCEP, and brand passion play crucial roles in the formation of brand addiction. These results provide conceptual frameworks to further understand the internal mechanism of brand addiction. Thus, this study supports the distinct treatment of the relationships between consumers and brands based on data analysis.

Our findings also confirm that SEBs and SUSCEP are positively related to HBP and OBP. A consumer who perceives brands to enhance his or her identify may gradually develop harmonious or obsessive passion for those brands. As anticipated, and in line with Swimberghe et al. (2014), SEBs have positive impact on harmonious passion and obsessive passion. Meanwhile, considering that most consumers needs to identify with others or meet their expectations (Kurt et al., 2011), it is understandable that SUSCEP has positive effects on both HBP and OBP. This lends further support to the argument of Belk et al. (2003) that brand passion tends to be more prominent under interpersonal influence. Our findings show how the psychological effects generated by consumers’ SUSCEP can promote HBP and OBP.

Third, after the analysis of the mediating effect of HBP and OBP, the research further reveals the influence path of SEBs and SUSCEP on consumer brand addiction, and the empirical results also effectively prove that the structural equation model proposed by the author is feasible. The study found that HBP and OBP have a certain mediating effect, which shows that HBP and OBP play key roles in the effectiveness of SEBS and SUSCEP in strengthening brand addiction. In view of the fact that the research about brand addiction is still at the first stage, on the one hand, the findings will help to encourage experts and scholars to continue to carry out special research on brand addiction, on the other hand, it can also significantly mobilize scholars’ interest in the joint effect of brand addiction and other forms of consumer brand relationship, so as to expand the research scope of this subject.

In addition, by clarifying the formation mechanism of brand addiction, this research helps to explain its positive and negative outcomes. To date, some research on brand addiction has suggested that addictive relationships with brands are destructive and progressive (e.g., Fournier and Alvarez, 2013), but the latest evidence shows brand addiction may not cause detrimental effects, partly because of its positive correlation to self-esteem (Cui et al., 2018). Furthermore, Mrad et al. (2020) believes that brand addiction is like a double-edged sword, which may reduce or improve the happiness of consumers, revealing not all patterns of addictive behavior are just pathological. Our study finds that both HBP and OBP are positively associated with brand addiction. We believe our research supports the assertions of Mrad et al. (2020) about brand addiction. Indeed, the desire to get it and willing to put in the effort can generate inner satisfaction in consumers, which is conducive to the autonomous internalization process of consumer–brand identification and the generation of positive brand addiction. However, if the relationship with the brand is not controlled by the consumer, OBP can form, leading to the emergence of negative brand addiction. Our findings indicate that HBP and OBP appear to have similar effects on brand addiction.

Finally, this study addresses the call by Swimberghe et al. (2014) to explore the relationship between OBP and brand addiction, and it supplements prior studies (e.g., Mrad et al., 2020) on the consumer–brand relationship continuum by adding two key variables that impact on brand addiction—i.e., HBP and OBP. The results could help marketing scholars or clinical psychologists to deepen the understanding of addiction and find out the potential adverse effects.

### Managerial Implications

The study has some implications for marketers, particularly those who want to establish a highly emotional consumer–brand relationship. Our findings can inform brand managers on what factors to prioritize to generate more intense consumer–brand connections, thus helping them to develop a scientific and efficient brand strategy, and point out the direction for improving the efficiency of customer relationship management. Marketers can use our results to classify consumers and segment their markets according to different levels of consumer–brand relationship intensity.

By exploring the key antecedents of brand addiction, the study can help marketers to clarify key roles of SEBs and SUSCEP. Managers should consider customers’ desire for self-expression and their need to connect with others. When some customers show their personal identity or values, they often give priority to the brand that suits them instead of outstanding in the market. Brand managers can cater to these tendencies by providing a forum where customers can express themselves and satisfy their desire for self-confirmation but also form connections with other similar consumers and be affected by others’ brand selections. Brand managers should ensure that members are able to freely interact in a friendly online community, which will enable marketers to examine perceptions and emotions toward their brands in real time.

Despite the significant contribution of SUSCEP, SEBs have a greater effect on OBP and HBP. This suggests that emphasizing self-expression in brand positioning is more important to enhance the consumer–brand relationship. Brand managers seeking to build more intense connections with their brands and engender passion for their products should focus on the self expression of the brand. For instance, marketing promotion may encourage consumers to take the brand as a breakthrough, and take the initiative to form close contact with it, so that their own needs can be expressed and transmitted. Furthermore, managers can seek to highlight the distinctive personality of a brand from multiple angles (such as design, brand name, advertising, brand endorser), allowing consumers to judge intuitively which brand suits them and expresses their identity and value. Companies can build stronger brands by identifying the aspects of customers’ behaviors and lifestyles most relevant to their need for self-expression.

In addition, the mediating roles of OBP and HBP identified in this research underscore the need for a “passion branding” strategy. Albert et al. (2013) argued that if certain brands play important roles in the process of a consumer’s identity acquisition, then his/her emotion toward the brands will be stronger. Additionally, brand managers can seek ways for consumers to gain positive social recognition and self-worth through their brands, thereby promoting brand emotion. Activating these kinds of intense emotional responses is important to improve a brand’s competitive position.

Empirical evidence attests to the nature and complexity of brand addiction. The views obtained can provide certain reference for brand managers to maintain the relationship between brands and consumers. A key question, however, is how brand managers should respond to the negative consequences of brand addiction. As addiction to brands may cause negative consequences at times, brand managers should adopt different marketing strategies. For example, for consumers who have the ability to balance the relationship between their own life and brand passion, brand managers can continuously try to deepen their relationship with brands, hence helping them to experience positive brand addiction. However, for consumers whose brand addiction becomes dominant and causes an imbalance with other areas of life, brand managers can design small packaging or enforce purchase limitations to reduce the psychological pressure.

### Limitations and Future Research

The study still has many deficiencies, so it needs to be further improved in the future. First, the research payed attention to the fashion context, with the respondents from only one country. Therefore, the results of this study may not be directly applied to other situations. Future research can extend this investigation to other countries and investigate differences among brand categories. Second, the study was based on social identity theory. Future research can apply other theoretical models to explore variables related to brand addiction. Third, the scope of this research was limited to the drivers or antecedents of brand addiction, which were analyzed to understand the formation mechanism and motivations for brand addiction. In future research, the influence of consumer brand addiction on subsequent behaviors can be analyzed. Finally, although our research considered brand passion and responded to the call of Mrad and Cui (2017) to explore the role of brand addiction within an integrated framework of consumer–brand relationships, there is a need to consider other factors (e.g., compulsive buying and other types of addictive behaviors) to further deepen the understanding of brand addiction.

## Data Availability Statement

The original contributions presented in the study are included in the article/supplementary material, further inquiries can be directed to the corresponding author/s.

## Ethics Statement

The studies involving human participants were reviewed and approved by the University of Commerce. All participants were consumers familiar with a certain brand finding through the Harbin Mingyue Market Research Consulting Co., Ltd. using questionnaire. They verbally agreed to participate in this study and returned the questionnaire within a fixed time.

## Author Contributions

SB and YYi: conceptualization. YYi and SW: methodology. SW: software. SW and RW: formal analysis and investigation. RW: resources. YYi: writing-original draft preparation. YYi and YYu: writing-review and editing. SB and YYu: supervision. SB: project administration and funding acquisition. All authors contributed to the article and approved the submitted version.

## Conflict of Interest

The authors declare that the research was conducted in the absence of any commercial or financial relationships that could be construed as a potential conflict of interest.

## Appendix

**TABLE A1:** Measures.

Construct	Items	Mean	SD
Inner self	This brand symbolizes the kind of person I really am inside.	3.83	0.97
	This brand reflects my personality.	3.93	0.94
	This brand is an extension of my inner self Source: Colliander and Dahlén (2011).	4.15	0.77
	This brand mirrors the real me.	3.79	0.99
Social self	This brand contributes to my image.	3.89	0.97
	This brand adds to a social “role” I play.	3.78	1.03
	This brand has a positive impact on what others think of me.	3.74	1.00
	This brand improves the way society views me.	3.48	1.18
Susceptibility to interpersonal influence	I achieve a sense of belonging by purchasing the same products and brands that others purchase.	3.69	1.05
	If I want to be like someone, I often try to buy the same brands that they buy.	3.09	1.25
	I often identify with other people by purchasing the same products and brands they purchase.	3.38	1.15
Harmonious brand passion	This activity allows me to live a variety of experiences.	3.93	0.84
	The new things that I discover with this activity allow me to appreciate it even more.	4.00	0.87
	This activity allows me to live memorable experiences.	3.72	1.00
	This activity reflects the qualities I like about myself.	4.04	0.82
	This activity is in harmony with the other activities in my life.	3.90	0.95
Obsessive brand passion	I cannot live without it.	3.32	1.25
	The urge is so strong. I can’t help myself from doing this activity.	3.56	1.17
	I am emotionally dependent on this activity.	3.40	1.17
	I have a tough time controlling my need to do this activity.	3.25	1.27
	I have almost an obsessive feeling for this activity.	3.14	1.34
Brand addiction	I try very hard to get everything from my favorite brand.	2.99	1.39
	I often fail to control myself from purchasing products of my favorite brand.	3.07	1.37
	I often find myself thinking about my favorite brand.	3.73	1.08
	I tend to give up some life activities and duties, such as the occupational, academic, and familial, to fulfill some activities related to my favorite brand.	2.80	1.29
	I tend to allocate a certain portion of my monthly income to buy the products of my favorite brand.	3.44	1.13
	I usually remember tenderly the previous experiences with my favorite brand.	3.79	0.95
	I experience a state of impatience immediately before I can get hold of the products of my favorite brand.	3.78	1.09
	I follow my favorite brand’s news all the time.	3.82	1.08
	I usually plan when the next purchase of my favorite brand will be.	3.88	1.08
	I would invest my money in some way to support my favorite brand.	3.78	1.14
